# Mass blood survey for malaria: pooling and real-time PCR combined with expert microscopy in north-west Thailand

**DOI:** 10.1186/1475-2875-11-288

**Published:** 2012-08-21

**Authors:** Kanungnit Congpuong, Aungkana SaeJeng, Rungniran Sug-aram, Supannee Aruncharus, Ampai Darakapong, Steven R Meshnick, Wichai Satimai

**Affiliations:** 1 Bureau of Vector-Borne Diseases, Ministry of Public Health, Nonthaburi, Thailand; 2 Office of Disease Prevention and Control, #10, Chiang Mai, Thailand; 3 Department of Epidemiology, University of North Carolina, Chapel Hill, North Carolina, 27599-7435, USA; 4 Faculty of Science & Technology, Bansomdejchaopraya Rajabhat University, Bangkok, Thailand

## Abstract

**Background:**

Asymptomatic carriage of *Plasmodium falciparum* and *Plasmodium vivax* is common in both low-and high-transmission settings and represents an important reservoir of infection that needs to be targeted if malaria elimination is to succeed.

**Methods:**

Mass blood examinations (475 individuals) were conducted in two villages in Mae Hong Son, an area of endemic but low-transmission malaria in the north-west of Thailand. The microscopist at the local malaria clinic did not detect any infections. Pools of four samples were screened by real-time PCR; individual members of all of the positive pools were then re-examined by expert microscopy and by a second species-specific PCR reaction.

**Results:**

Eight subjects were found to be positive by both PCR and expert microscopy and one was found to be positive by PCR alone. The slides contained asexual stage parasites of *P. vivax, P. falciparum* and *Plasmodium malariae,* but no gametocytes. The local clinic was notified within two to eight days of the survey.

**Conclusion:**

A combination of pooling, real-time PCR and expert microscopy provides a feasible approach to identifying and treating asymptomatic malaria infections in a timely manner.

## Background

Active case detection (ACD) is a key component of malaria surveillance programmes. As early as 1848, active surveillance for splenomegaly was used as an indicator of malaria prevalence [1]. ACD programmes now employ microscopy and/or rapid diagnostic tests. In Thailand, three types of ACD are used: contact tracing (“Case Investigation Survey”), high-risk group screening (“Special Case Detection”), and mass blood examination.

ACD may have an even greater impact using PCR, which detect large numbers of infections that were previously undetected by microscopy (“submicroscopic infections”) [2-10]. Asymptomatic carriers could represent an important reservoir [3,9,11] and thus be of particular relevance to elimination efforts [12]. Historically, infections were identified by microscopy, however, a myriad of studies has shown that PCR is much more sensitive.

A high-throughput real-time PCR protocol for detecting parasites and determining their species was previously reported [7,13]. Pools of four samples were screened first using PCR primers that recognize all malaria species; individuals in positive pools were then screened with species-specific primers. Pooling led to substantial reductions in cost and effort for a Case Investigation Survey/Special Case Detection study in Bo Rai District, Trat Province, Thailand [7]. PCR detected four infections while the local microscopist detected none. Three of the four PCR-positive cases were later confirmed by an expert microscopist. The current paper shows that pooled real-time PCR coupled with expert microscopy enhances sensitivity of detection in mass blood examinations.

## Methods

Two villages in Mae Hong Son were selected for mass blood examinations. Mae Hong Son is in north-west Thailand near the Myanmar border. Hueypo is a village of 657 residents, screened. In 2011, three *Plasmodium vivax* and five *Plasmodium falciparum* infections were found by passive case detection. Maesuad is a village of 242 residents. In 2011, four *P. vivax* and two *P. falciparum* infections were found by passive case detection. Thus, both villages were considered endemic for malaria.

On 13 and 14 March, 2012, a team from the Bureau of Vector Borne Disease (BVBD) visited schools and conducted house-to-house surveys in both villages. Of 657 residents, 362 (55%) were tested in Hueypo and 113 of 242 residents were tested in Maesuad (47%). Finger-prick blood samples were taken and used to prepare thin and thick smears as well as dried blood spots. Microscope slides were examined on 15 March by the microscopist at the local malaria clinic.

DNA was extracted from dried blood spots, pooled, and PCR-analyzed as previously described [7]. In brief, the dried blood spots were brought on the next day to a laboratory in nearby Chiang Mai, where DNA was extracted. Pools of four were tested by the all-*Plasmodium* primer-probe set as previously described except an ESCO Swift Spectrum 48 Real Time Thermal Cycler was used. Pools representing all of the samples from Maesuad were tested on 15 and 16 March. Due to time constraints, the team had to leave Chiang Mai. The remaining samples were brought to the BVBD laboratories in Nonthaburi where all of the remaining reactions were carried out on 19 and 20 March using a Bio-Rad CFX96™ Real-Time PCR System. For each positive pool, the individual samples were tested by species-specific PCR. In addition, thick smears for each individual in a positive pool were examined by a Level 1 expert microscopist before and after the results of the species-specific PCR were known. None of the PCR-negative slides were re-examined. The malaria clinic was notified by phone of all microscopy and PCR positive samples and the individuals were treated according to national guidelines. Statistical significance was determined using JMP ver 8.0 (SAS Institute, Cary, NC, USA).

## Results

Of 475 subjects, none was found to be malaria-positive by the microscopist at the local malaria clinic. Eight pools were found to be positive by PCR. Of the 32 subjects represented by these pools, nine were found to be malaria positive by PCR using pan-species primers (Table 1), for a prevalence of 1.9%. All of these samples were also positive on a second PCR reaction using species-specific PCR primers. One *P. falciparum* infection, two *P. vivax* infections, three *Plasmodium malariae* infections and three *P. falciparum*/*P. malariae* co-infections were found.

**Table 1:** PCR-positive malaria infections

**Patient**	**Village**	**Age (yrs)**	**Sex**	**Occupation**	**Species (by PCR)**^**a**^	**Expert microscopy**	**Time-to notification**
141	Maesuad	16	F	Agric. worker	PF	1 PF ring	2 days
34	Maesuad	4	M	Child	PV	3 PV amoeboid forms	2 days
107	Maesuad	12	M	Child	PV	3 PV amoeboid forms	2 days
144	Maesuad	38	M	Labourer	PM	Negative^b^	2 days
152	Maesuad	3	M	Child	PF/PM co-infection	1 ring, could not speciate	2 days
110	Hueypo	16	M	Child	PF/PM co-infection	1 ring, could not speciate	6 days
391	Hueypo	50	F	Agric. worker	PF/PM co-infection	Negative/Positive^c^	8 days
88	Hueypo	14	F	Child	PM	1 ring, could not speciate	6 days
390	Hueypo	18	F	Agric. worker	PM	1 ring, could not speciate	8 days

^a^*PF*: *P. falciparum*; *PV*: *P. vivax*; *PM*: *P. malariae.*

^b^ Quality of blood film was poor.

^c^ Negative when examined before species-specific PCR; positive when examined afterwards.

Expert microscopists examined slides from the eight positive pools before the second-round PCR and identified seven infections. Very scant parasites were found in all slides; often only a single parasite was observed. All seven were subsequently found to be PCR-positive. In four of the seven cases, the expert microscopist found a single ring which could not be accurately speciated. For the remaining three cases (two *P. vivax* and one *P. falciparum*), the expert microscopist and PCR agreed.

The second-round, species-specific PCR detected two additional cases not initially detected by expert microscopy (nos. 144 and 391). After the results of the PCR were known, the expert microscopists re-examined the two discordant slides. A single parasite was found on the slide for patient 391. No parasites were seen on the slide for patient 144, but the quality of the film was poor.

All of the *P. falciparum* and *P. malariae* parasites that were observed were ring-stage. The *P. vivax* infections included rings and trophozoites. No gametocytes were seen.

Both microscopy and PCR results were reported to the local malaria clinic in a timely fashion. PCR was started on the Maesuad samples one day after the survey. The results were phoned in to the malaria clinic one day later. The samples from Hueypo were started six days after the survey. Of these, three positive pools were found. The malaria clinic was notified eight days after the survey. In summary, the local malaria clinic was notified between two and eight days after the blood draw (mean = 4 days, median = 2 days).

Table 2 compares infected and uninfected individuals. Infected individuals tended to be younger, but the difference was not statistically significant (Wilcoxon/Kruskal-Wallis test). There was no significant difference in the ratio of males to females between infected and uninfected individuals. The prevalence of malaria was significantly higher in Maesuad (4.4%) than Hueypo (1.1%) (p = 0.04, Fisher’s two-tailed exact test). All were Thai except for patient 110.

**Table 2:** Characteristics of infected and uninfected subjects

**Characteristic**	**Uninfected (466)**	**Infected (9)**
Median age (IQR)	25 (10-45)	16 (12-18)
Percent male	44	56
Maesuad	108	5 (4.4%)
Hueypo	358	4 (1.1%)

One of the patients who was positive by both PCR and expert microscopy (no. 88) appeared to clear parasitaemia spontaneously. When the field staff visited this patient six days after screening, she was found to be negative by both PCR and by expert microscopy.

## Discussion

In this paper, real-time PCR of sample pools, followed by species-specific PCRs and expert microscopy, has been shown to be an effective surveillance tool. Almost 2% of individuals screened by mass blood examination were positive by PCR, but were microscopy negative when the slides were examined by the local microscopist. Expert microscopists detected parasites in eight of the nine PCR-positive subjects. Thus, a classical mass blood examination would have missed a substantial number of infections.

In this study, expert microscopy was performed on slides from individuals in the positive pools before the second round PCR was done. This enabled some results to be reported the same day the pooled PCR results were obtained. This raises the possibility that PCR and pooling can be used to reduce microscopy time. Local microscopists usually spend only about 10 minutes examining a slide, but, as described above, the sensitivity of local microscopy is low. The sensitivity of expert microscopy is much higher, partially because about 30 minutes are required for an examination. If an expert microscopist were to examine all 475 slides, it would take ~237.5 hours. However, using the results of the pooled PCR as a guide, the experts had to examine 32 slides (eight pools), taking ~16 hours. This is much less than the time spent by the local microscopist examining all 475 slides (~80 hours). After pooled PCR and expert microscopy, results were reported as quickly as 48 hours after blood was drawn. Thus, a combination of PCR pooling and expert microscopy might be an appropriate surveillance tool.

The study has two limitations. First, expert microscopy was only performed on PCR-positive pools, so the true sensitivity and specificity of real-time PCR cannot be calculated. Second, only about half of the village residents participated in the survey, so the true malaria prevalence may be different.

Of particular interest was the observation that none of the infected individuals contained gametocytes in their blood. This was also true in the previous study in Trat (Rogawski et al., unpublished results). This has implications for understanding malaria transmission. In a previous study in Mae Hong Son [14], infectivity was closely associated with the presence of gametocytes in the blood. The results presented here suggest that the prevalent asymptomatic infections may not be major infectious reservoirs at the time of the survey. However, it is possible that many of these asymptomatic infections represent early stage infections. Thus, the true value of PCR-based surveillance might be in early detection and prevention of disease and subsequent mosquito infectivity. On the other hand, it is also possible that many of these infections might clear parasitaemia, just as patient 88 appeared to do.

In summary, this paper shows that pooling and real-time PCR, combined with expert microscopy is rapid and cost-effective and is superior to local microscopy in detection of infections. It might serve an important role in efforts to eliminate malaria in low-transmission settings.

## Competing interests

The authors declare that they have no competing interests.

## Authors' contributions

AS, RS, SA and AD obtained the dried blood spots, extracted DNA, ran the PCR’s. KC and AS are expert microscopists and did blood examinations KC, SM and WS participated in analyzing the data and writing the manuscript. All authors read and approved the final manuscript
